# Physiological and biochemical changes in Moroccan barley (*Hordeum vulgare* L.) cultivars submitted to drought stress

**DOI:** 10.1016/j.heliyon.2023.e13643

**Published:** 2023-02-10

**Authors:** Mohamed Ferioun, Nassira Srhiouar, Said Bouhraoua, Naïma El Ghachtouli, Saïd Louahlia

**Affiliations:** a Natural Resources and Environmental Laboratory. Taza Polydisciplinary Faculty, Sidi Mohamed Ben Abdellah University, Fez, Morocco; b Microbial Biotechnology and Bioactive Molecules Laboratory, Sciences and Technology Faculty, Sidi Mohamed Ben Abdellah University, Fez, Morocco

**Keywords:** *Hordeum vulgare*, Barley, Drought stress, Drought tolerance, Physiological parameters

## Abstract

Barley (*Hordeum vulgare* L.) is the second most consumed and cultivated cereal by the Moroccan population. However, it is predicted that frequent drought periods, caused by climate change, can cause problems in plant growth. Thus, the selection of drought-tolerant barley cultivars is essential to ensure the security of barley's needs. We aimed to screen drought stress tolerance in Moroccan barley cultivars. We tested the drought tolerance of nine Moroccan barley cultivars (‘Adrar’, ‘Amalou’, ‘Amira’, ‘Firdaws’, ‘Laanaceur’, ‘Massine’, ‘Oussama’, ‘Taffa’, and ‘Tamellalt’) based on physiological and biochemical parameters. Drought stress was applied by maintaining field capacity at 40% (90% for the control), and plants were randomly arranged in a greenhouse at 25 °C under natural light conditions. Drought stress decreased relative water content (RWC), shoot dry weight (SDW), and chlorophyll content (SPAD index), but significantly increased electrolyte leakage, hydrogen peroxide, malondialdehyde (MDA), water-soluble carbohydrates, and soluble protein contents, as well as catalase (CAT) and ascorbate peroxidase (APX) activities. High levels of SDW, RWC, CAT, and APX activities were recorded in ‘Firdaws’, ‘Laanaceur’, ‘Massine’, ‘Taffa’, and ‘Oussama’, which can be interpreted by high drought tolerance. On the other hand, ‘Adrar’, ‘Amalou’, ‘Amira’, and ‘Tamellalt’ showed higher values of MDA and H_2_O_2_ content, which can be linked with drought sensitivity. Physiological and biochemical parameter changes are discussed in terms of barley's tolerance to drought. Tolerant cultivars could be a good background for barley breeding in areas known for the alternative of long dry spells.

## 1 Introduction

Cereals in their growth period face various biotic and abiotic stresses, apart or combined, which markedly impact morphological and physiological parameters [[1], [2], [3]]. Climate change-induced global warming may cause increasingly severe and frequent droughts and intensify abiotic impacts on agricultural productivity in various parts of the world [4]. It's expected that these environmental conditions could cause serious plant growth problems for more than 50% of arable areas by 2050 [5].

In terms of areas farmed, barley is the fourth most significant crop in the world, after maize, wheat, and rice [6]. The water shortage greatly reduces its yield potential and yearly productivity compared to the other grains described above [7]. Numerous studies worldwide showed that water deficit induces many changes in barley. Morphologically, drought induces decreases in roots and shoots dry weight [[8], [9], [10]]. Physiologically, water deficit decreases the relative water content in leaves [[11], [12], [13], [14]], and induces cell membrane deterioration [12,15]. Previous research has clearly demonstrated that water withdrawal causes significant damage to the photosynthesis apparatus [[16], [17], [18]], as well as nutrient uptake [19]. It is also widespread that physiological damage resulting from drought stress induces the oxidative stress resulting from reactive oxygen species generation (ROS) [20,21]. And increases the synthesis of stress hormones like abscisic acid (ABA) [19], and affects grain yield [3]. Indeed, a reduction of grain yield indexes was noted in different studies [1,22,23]. Thus, the study of physiological and biochemical parameters in barley under drought stress is very important regarding the understanding of the reaction of different barley cultivars against abiotic stress.

Intraspecific variability between barley varieties, cultivars, genotypes, and lines regarding the adaptation to drought conditions is widely spread in the literature [9,17,[24], [25], [26]]. The study of genetic basis of barley drought tolerance describes genes related to traits involved in drought stress tolerance. Many quantitative traits loci were found especially in tolerant genotypes [19]. The difference in the expression of these genes influences plants’ physiology, makes barley plants able to keep high cell turgor, maintains photosynthesis efficiency, modulates osmotic statue by the adaptation of osmolytes content, and preserves membrane cell steadiness [19]. Based on this approach, we think that the screening of cultivars showing high physiological tolerance to drought stress could lead those described as having a powerful genetic pool to tolerate stressful conditions. In literature, numerous methods were described to evaluate physiological tolerance in various plants, relative water content (RWC) is measured to evaluate the ability of leaf plants to keep cells turgor [14,27], electrolyte leakage % is linked with cell membrane deterioration [28], chlorophyll content for the photosynthesis apparatus steadiness [10], proline, soluble sugars, and soluble proteins contents to describe the ability of plants to adjust the metabolism for osmotic statue adaptation [19,28], hydrogen peroxide and malonaldehyde (MDA) contents for the oxidative status [29]. For the later, the activity of antioxidant enzymes could also be measured to record if plants can modulate his enzymes machine under stressful conditions [30].

Based on the above, the reaction to drought stress in barley varies from one cultivar to another because of the properties of each cultivar, and the description of physiological and biochemical traits in different Moroccan cultivars might be an important section for having a comprehensive and clear view concerning the adaptation of Moroccan barley cultivars to drought stress. To the best of our knowledge, such studies describing physiological and biochemical behavior of Moroccan barley cultivars are very limited. To ensure food quantity, quality, and security, the selection and description of tolerant cultivars becomes highly required. Thus, this work aims to asses nine Moroccan cultivars of barley (*Hordeum vulgare* L.) for their tolerance to drought stress through the study of their physiological and biochemical responses to drought conditions.

## 2 Material and methods

### 2.1 Determination of field capacity of pot soil

Pots were prepared using soil, sable, and peat (1:1:1) (3.75 kg/pot) in order to measure field capacity (FC), the soil was saturated with water, and the weight of the soil when drainage ceased (24 h) was noted. The gravimetric technique was used to determine the moisture content of the soil. Prior to and following oven drying for 72 h, soil samples were weighed. The results were then divided by the weight of the oven-dry soil [24].

### 2.2 Growth conditions

Homogenous barley seeds (*Hordeum vulgare* L.) of nine Moroccan cultivars (Table 1) (‘Oussama’, ‘Laanceur’, ‘Adrar’, ‘Massine’, ‘Amira’, ‘Amalou’, ‘Taffa’, ‘Firdaws’, ‘Tamellalt’) were obtained of seeds stock of RNE-lab at polydisciplinary faculty of Taza and the Moroccan National Institute of Agricultural Research (INRA).

**Table 1:** Description of the barley cultivars used in this study [31].

Official name	Row type	Spring/winter type	Hulled/hulless	Earliness of maturity	Year of release
‘Adrar’	2 rows	Spring Type	Hulled	Medium type	1998
‘Firdaws’	6 rows	Winter type	Hulled	Medium type	1998
‘Amalou’	6 rows	Winter type	Hulled	Early type	1997
‘Amira’	6 rows	Winter type	Hulled	Medium type	1996
‘Oussama’	6 rows	Winter type	Hulled	Medium type	1995
‘Taffa’	6 rows	Winter type	Hulled	Medium type	1994
‘Massine’	6 rows	Winter type	Hulled	Medium type	1994
‘Laannaceur’	6 rows	Winter type	Hulled	Medium type	1991
‘Tamellalt’	2 rows	Spring Type	Hulled	Medium type	1984

The seeds surface was disinfected in sodium hypochlorite (10%) bath and rinsed with distilled water. Seeds were sown in Petri dishes containing wet Whatman paper and incubated at 25 °C up to the coleoptiles reaches 1 cm length. Then, seeds were placed in plastic pots. Three weeks after sowing (third leaf stage), two plants were maintained in each pot, and drought stress was applied by maintaining water content in stressed pots at 40% of FC. For control pots, FC was maintained at 90%. Pots were arranged randomly in a greenhouse under natural light, the temperature was maintained at 25 °C and humidity at 75% up to the eighth week when the third node of barley plants was detectable (GS33 growth stage based on Zadoks decimal code).

### 2.3 Determination of shoot dry weight (SDW)

Shoot part of barley plants was harvested and dried at 60 °C for 72 h, then, the weight of each plant was noted and considered as shoot dry weight (SDW).

### 2.4 Determination of plant physiological and biochemcal parameters

Eight weeks after sowing, leaf samples were harvested and stored at −20 °C up to analysis.

#### 2.4.1 Relative water content (RWC)

Leaves Relative Water Content (RWC) was estimated following the method described by Sarker and Oba [32] using formula:

$$RWC\;\left(\%\right)=\frac{Fresh\;weight-Dry\;weight}{Turgor\;weight-Dry\;weight}\times 100$$

#### 2.4.2 Proline content

Proline content was determined following the method described by Sarker and Oba [33]. Briefly, 200 mg of leaf sample was homogenized with 4 mL of sulfoscalicylic acid (3%) and centrifuged at 10 000 rpm for 10 min 2 mL of supernatant was mixed with 2 mL of glacial acetic acid and 2 mL of Ninhydrin reagent was added to the mixture. The set was incubated 1 h at 100 °C. Proline was extracted in toluene and the absorbance was measured at 520 nm. Proline content was measured using a standard curve of l-proline.

#### 2.4.3 Electrolyte leakage (EL)

Electrolyte leakage (EL) was evaluated using the technique of Sarker and Oba [34]. Briefly, leaf samples (500 mg) were emerged in distilled water overnight at room temperature, then, electrical conductivity was noted (EC1). The samples were incubated at 95 °C for 10 min and the electrical conductivity was measured (EC2). EL was calculated using the formula:

EL% = (EC1/EC2)*100

#### 2.4.4 SPAD index (chlorophyll content)

Chlorophyll content was measured using Chlorophyll meter SPAD-502plus (SPAD KONICA MINOLTA, made in Japan). In barley, a significant correlation between SPAD value and chlorophyll content was noted [35].

#### 2.4.5 Hydrogen peroxide (H_2_O_2_) content and lipid peroxidation

250 mg of leaf sample was homogenized with 5 mL of Trichloroacetic acid (TCA) 0.1%. The supernatant was taken after centrifugation (10 000 rpm/5 min) and considered as the extract of H_2_O_2_ and malondialdehyde (MDA).

To quantify H_2_O_2_ content, 0.2 mL of extract was added to 0.8 mL of phosphate potassium buffer (10 mM, pH7), then, 1 mL of KI (1 M) was added to the mixture. The set was incubated 10 min at room temperature, and the absorbance was measured at 390 nm (Spectrophotometer JASCO V-730, made in Japan). H_2_O_2_ content was measured using a standard curve of H_2_O_2_ [33].

Lipids peroxidation was evaluated by quantifying leaf MDA content. It was estimated using the method described by Sarker and Oba [33]. Briefly, 1 mL of the extract was added to 4 mL 20% TCA containing Thiobarbituric acid 0.5%. The set was heated at 95 °C for 10 min. After centrifugation (10 000 rpm/5 min), the absorbance was noted at 532 nm and 600 nm (Spectrophotometer JASCO V-730, made in Japan). MDA content was estimated using the extinction coefficient of MDA at 532 nm which is 155 mM^−1^ cm^−1^.

#### 2.4.6 Total soluble sugars content (TSS)

The extract of total soluble sugars (TSS) was prepared following the method of Erice et al. [36] Briefly, 100 mg fresh leaf was mixed with 5 mL of phosphate potassium buffer (50 mM, pH 7.5) and centrifuged 15 min at 10 000 rpm. The supernatant was used as the extract of TSS.

TSS content was measured using the method of Yemm and Willis [37]. Briefly, 0.1 mL of supernatant was added to the anthrone reagent, and the mixture was heated at 90 °C for 10 min, and the absorbance at 625 nm (Spectrophotometer JASCO V-730, made in Japan). TSS was measured using a standard curve of d-glucose.

#### 2.4.7 Soluble proteins content

Leaf sample (200 mg) was homogenated on ice in 2 mL of refrigerated phosphate sodium buffer (100 mM, pH 7.5). The supernatant taken after centrifugation (8000 rpm/15 min) was considered as proteins extract. Soluble proteins content was determined photometrically (Spectrophotometer JASCO V-730, made in Japan) following the method described by Sarker et al. [38] using a standard curve of Bovine Serum Albumine (BSA).

#### 2.4.8 Enzyme activities

Around 500 mg of leaves samples was ground with 4 mL of 50 mM phosphate potassium buffer (pH 7) in an ice-cold mortar. The mixture was centrifuged at 20 000 rpm for 30 min at 4 °C. the supernatant was considered as the enzymes extract. Catalase (CAT) (EC: 1.11.1.6) activity was measured photometrically following the method described by Sarker and Oba [32]. The reaction was started by adding 50 μL of enzymes extract to the assay solution containing phosphate potassium buffer (50 mM, pH 7) and H_2_O_2_ (10 mM), the mixture was incubated 5 min at room temperature and the absorbance was measured at 240 nm, CAT activity was calculated using molar extinction coefficient ε = 43.6 M^−1^ cm^−1^. Ascorbate peroxidase (APX) (EC: 1.11.1.11) was determined photometrically using the method described by Sarker and Oba [32]. Briefly, the reaction was started by adding 50 μL of enzymes solution to the assay solution containing phosphate potassium buffer (50 mM, pH 7), H_2_O_2_ (1 mM), EDTA (0,2 mM), and ascorbic acid (0,5 mM), after 1 min at room temperature, the absorbance was measured at 290 nm. APX activity was calculated using molar extinction coefficient ε = 2.8 mM^−1^ cm^−1^.

### 2.5 Data analysis

All measurements were made in triplicates. Analysis of variances (ANOVA) was performed over drought stress and cultivars. Means were compared at 5% as a level of probability adopting the Fisher's Least Significant Difference's test (LSD) [39]. Correlation (Pearson) matrix was performed based on means values of parameters studied [40]. These statistical analyses were established using Minitab 18 statistical software.

Principal component analysis (PCA) was adopted in this current study as a multivariate statistical tool to represent the variability of parameters studied of different barley cultivars under drought stress [41]. Agglomerative hierarchical cluster analysis was carried out based on means values of parameters studied under drought stress through Euclidean distance to reveal the similarity between barley cultivars based on parameters studied [40] using XLSTAT software (XLSTAT Version 2016.02.28 451).

## 3 Results

### 3.1 Analysis of variances (ANOVA)

Table 2 displays the mean squares of the combined analysis of variances for SDW, RWC, SPAD, Electrolyte leakage (EL%), proline content, H_2_O_2_ content, MDA content, TSS content, total soluble proteins content, and CAT and APX activities in the leaves of the nine Moroccan barley cultivars exposed to drought stress. It's shown that cultivar, treatment and their interaction influenced the biochemical and physiological parameters studied significantly. However, treatment was the most important source of variability for all parameters studied except TSS and soluble protein contents (36 and 22% respectively). The effect of this factor is more than 80% for RWC and SPAD index, more than 65% for proline and MDA contents, and CAT and APX activities, and also little more than 50% for H_2_O_2_ content and EL%. For TSS content the major source of variability was cultivar factor with 60% of total variability, on the other hand, treatment by cultivar interaction is the important source of variability concerning soluble proteins content parameter with 42% of total variability (Table 2).

**Table 2:** Mean squares of the combined analyses of variance for the parameters studied in the nine barley cultivars.

Source	Df	SDW	RWC	EL	SPAD	Proline	H_2_O_2_	MDA	TSS	Proteins	CAT	APX
Cultivar (C)	8	0.63***	28.39***	211.47***	34.73***	36.04***	4.64***	80.33***	82.09***	1.65***	0.027***	4.04***
Treatment (T)	1	16.22***	201.28***	380.17***	532.04***	148.12***	7.89***	359.62***	49.03***	1.05***	0.068***	9.95***
Replicates	2	0.02	1.81	7.86	6.64	4.03	0.33	0.78	0.66	0.03	0.003	0.15
C*T	8	0.25***	13.41***	143.07***	15.92***	22.90***	1.76***	77.13***	3.43***	2.02***	0.004***	1.95***
Error	34	0.02	3.61	8.31	3.22	3.45	0.31	0.23	0.96	0.11	0.001	0.17
Total	53											

Df: Degree of freedom. *: significant at 0.05. **: significant at 0.01. ***: significant at 0.005. SDW: Shoot dry weight. RWC: Relative water content. EL: Electrolytes leakage. MDA: Malondialdehyde. TSS: Total soluble sugars. CAT: Catalase activity. APX: Ascorbate peroxidase activity.

### 3.2 Physiological characteristics of moroccan barley cultivars under drought stress

**Fig. 1:**
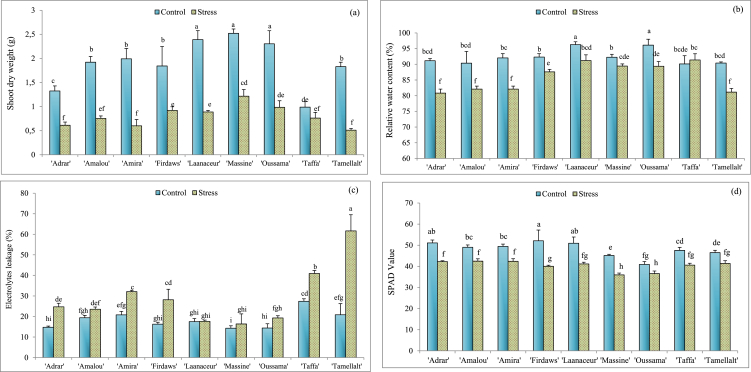
Means values of Shoot dry weight (a), relative water content (b), electrolytes leakage (c), and SPAD value (D) of barley varieties under optimal irrigated conditions (Control) and drought stress. Values indicate the mean (±SE) of n = 3. Scores with the same letter are not significantly different at P = 0.05.

**Fig. 2:**
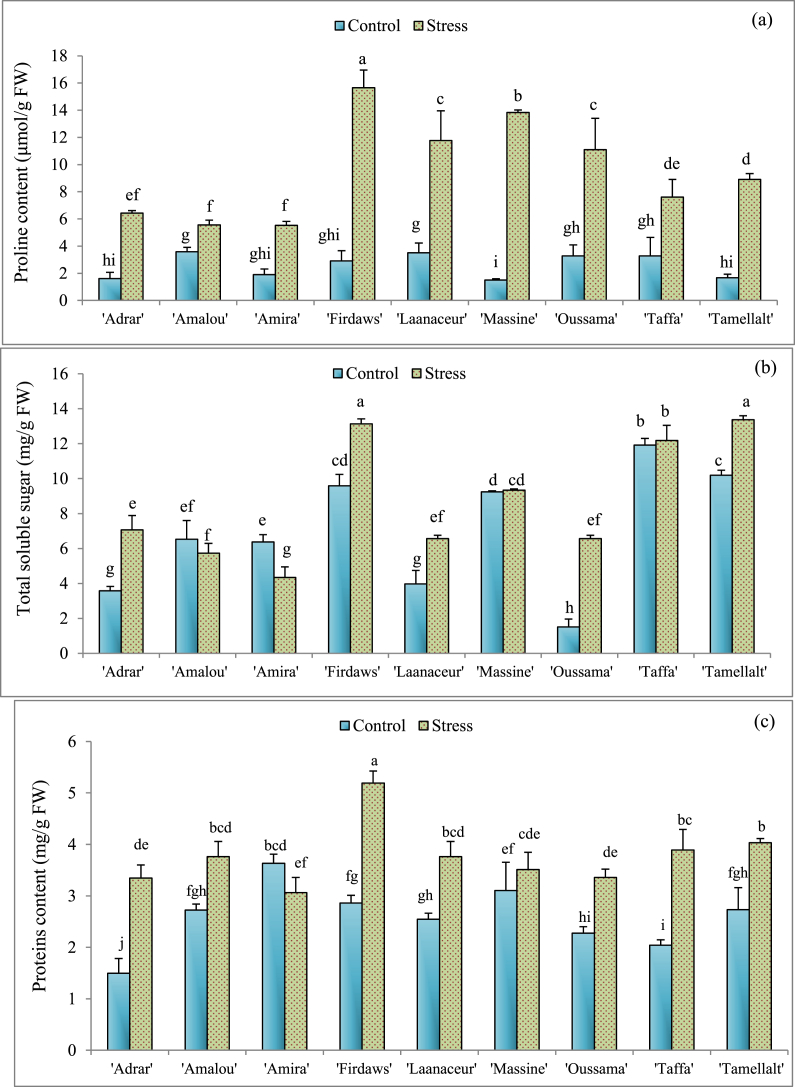
Means values of proline (a), total soluble sugars (b), and proteins (c) contents of barley varieties under optimal irrigated conditions (Control) and drought stress. Values indicate the mean (±SE) of n = 3. Scores with the same letter are not significantly different at P = 0.05.

**Fig. 3:**
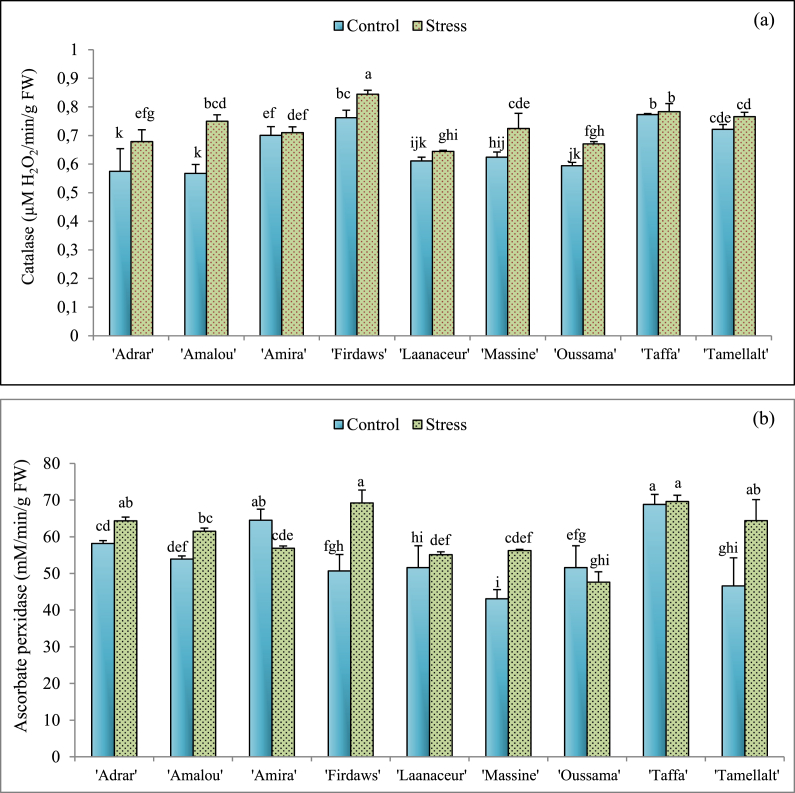
Means values of catalase (CAT) activity (a) and ascorbate peroxidase (APX) activity (b) of barley varieties under optimal irrigated conditions (Control) and drought stress. Values indicate the mean (±SE) of n = 3. Scores with the same letter are not significantly different at P = 0.05.

**Fig. 4:**
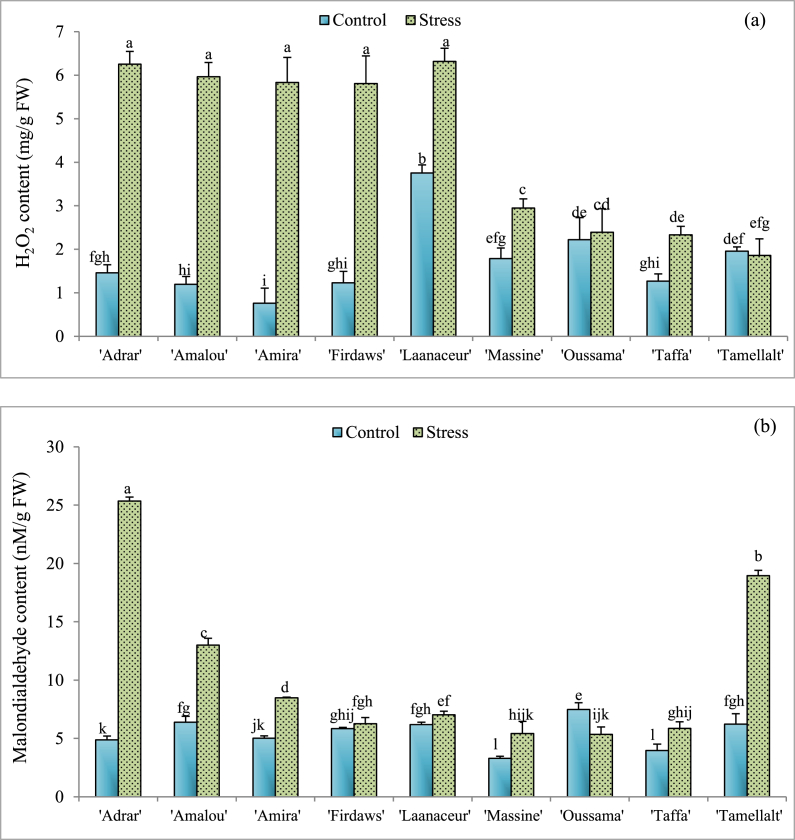
Means values of H2O2 (a) and Malondialdehyde (b) contents of barley varieties under optimal irrigated conditions (Control) and drought stress. Values indicate the mean (±SE) of n = 3. Scores with the same letter are not significantly different at P = 0.05.

The results shown in Fig. 1, Fig. 2, Fig. 3, Fig. 4 confirm the results of the analysis of variances. Significant differences were noted between cultivars in both irrigated (control) and stressed plants. Besides, it's clear that drought stress influenced most of the biochemical and physiological parameters studied for most of the cultivars. Our finding shows that drought stress reduced SDW (Fig. 1a) of barley cultivars studied compared to the control for all cultivars studied. It seems that ‘Amira’, ‘Amalou’, and ‘Tamellalt’ were the most influenced, where SDW was decreased by about 70% in stressed plants compared to the control. However, ‘Massine’ and ‘Oussama’ were less influenced, where SDW reduction was about 50%.

Other than ‘Taffa’, there was a significant reduction (P < 0.05) of RWC (Fig. 1b) in the leaves of the cultivars studied. When compared to the controls, the cultivars of ‘Adrar’, ‘Amalou’, ‘Amira’, and ‘Tamellalt’ had the most important decreases of RWC in their leaves, with reductions of 13.52%, 9.12%, 10.79%, and 10.24% respectively. The lowest reductions were noted in ‘Firdaws’ (5.13%), ‘Laanaceur’ (5.27%), ‘Massine’ (3.04%), and ‘Oussama’ (7.01%).

For electrolyte leakage (Fig. 1c), ‘Adrar’, ‘Amira’, ‘Firdaws’, ‘Tamellalt’, and ‘Taffa’ showed a significant increase (P < 0.05) of EL percentage under drought stress, with the highest score (61.65%) observed in ‘Tamellalt’ leaves. For ‘Amalou’, ‘Laanaceur’, ‘Massine’, and ‘Oussama’, there was no significant (P ≥ 0.05) difference in EL% between controls and stressed plant leaves.

All cultivars showed a significant (P < 0.05) decrease of SPAD values (Fig. 1d), where, ‘Adrar’, ‘Amalou’, ‘Amira’, ‘Firdaws’, and ‘Laanaceur’ were the most influenced (around 20% reduction). Besides, ‘Massine’, ‘Oussama’, ‘Taffa’ and ‘Tamellalt’ were less influenced (around 13% reduction). A significant increase in leaf proline content (Fig. 2a) was recorded in stressed plants compared to the control (well-irrigated). The cultivars that accumulated the most proline in leaves were: ‘Firdaws’ (81.43%), ‘Laanaceur’ (70.23%), ‘Massine’ (89.12%), and ‘Oussama’ (70.45%). Regarding Total Soluble Sugars (TSS), when compared to the controls, water deficit did not affect this parameter in ‘Amalou’, ‘Massine’, and ‘Taffa’ cultivars (Fig. 2b). However, ‘Firdaws’ and ‘Tamellalt’ showed the highest TSS content with increases of 26.98% and 23.75%, respectively, compared to the controls. According to the results obtained for soluble proteins (Fig. 2c), it was noted that drought stress increased soluble proteins content in the leaves of all cultivars, except in ‘Amira’ and ‘Massine’. In these cases, no significant difference between control and treatment was recorded (Fig. 2c).

Regarding enzyme activities, drought stress increased significantly (P < 0.05) CAT activity in ‘Adrar’, ‘Amalou’, ‘Firdaws’, ‘Massine’, and ‘Oussama’ cultivars' leaves (15.36%, 24.4%, 10.76%, 13.79%, and 11.34% respectively) (Fig. 3a). For the other cultivars, there is no statistical significant difference between control and stressed plants (Fig. 3a). Concerning APX activity, drought stress induced significant (P < 0.05) increases in this enzyme's activity in the leaf tissues of ‘Adrar’, ‘Amalou’, ‘Firdaws’, ‘Massine’, and Tamellalt. When compared to the controls, the recorded APX activities were 9.59%, 12.32%, 26.75%, 23.42%, and 27.68% higher in plants submitted to stress, respectively. The ‘Amira’ cultivar showed a significant decrease of APX activity in stressed plants, compared to the control. For the other cultivars, it seems that drought stress did not significantly affect the APX activity (Fig. 3b).

Except ‘Oussama’ and ‘Tamellalt’, all cultivars considered in this study had a significant increase (P < 0.05) of H_2_O_2_ content (Fig. 4a) under drought stress (‘Adrar’ (76.65%), ‘Amalou’ (79.95%), ‘Amira’ (86.95%), ‘Firdaws’ (78.79%), ‘Laanaceur’ (40.53%), ‘Massine’ (39.33%), and ‘Taffa’ (45.6%)). For MDA content, no significant difference was noted in ‘Firdaws’ and ‘Laanaceur’ between control and stressed plants (Fig. 4b). However, there was a significant increase (P < 0.05) in MDA content in the leaves of the other cultivars. The highest increases were 80.79% and 67.17% for ‘Adrar’ and ‘Tamellalt’, respectively, compared to the controls. For ‘Oussama’, a significant decrease (P < 0.05) of MDA content was recorded under drought stress.

### 3.3 Correlation among the biochemical and physiological parameters studied

Table 3 shows the correlation matrix among the physiological parameters studied under drought stress. Positive and negative correlations were noted. Our results showed that the SDW was correlated significantly (P < 0.05) and positively with RWC and proline content, and negatively with EL%, SPAD index, and MDA content. RWC was correlated negatively and significantly (P < 0.05) with MDA content. Regarding the results recording TSS content, this parameter was positively correlated with TSS content and CAT activity. CAT activity was positively and significantly (P < 0.05) correlated with total soluble proteins content and APX activity, and negatively with H_2_O_2_ content.

**Table 3:** Correlation matrix (Pearson) among parameters studied under drought stress.

Variables	RWC	EL	SPAD	Proline	H_2_O_2_	MDA	TSS	Proteins	CAT	APX
SDW	**0.730**^a^	**−0.694**^a^	−0.847***	**0.739**^a^	−0.153	**−0.686**^a^	0.009	0.117	−0.061	−0.397
RWC		−0.386	−0.614	0.610	−0.280	−0.797**	0.194	0.193	−0.047	−0.200
EL			0.383	−0.286	−0.468	0.360	0.603	0.226	0.469	0.526
SPAD				**−0.671**^a^	0.568	0.559	−0.151	0.029	0.087	0.491
Proline					−0.149	−0.513	0.460	0.590	0.228	−0.087
H_2_O_2_						0.195	−0.514	0.048	−0.872***	0.076
MDA							−0.016	−0.165	−0.130	0.309
TSS								**0.742**^a^	**0.744**^a^	0.666
Proteins									**0.780**^a^	0.618
CAT										**0.766**^a^

a Significant at 0.05. **: significant at 0.01. ***: significant at 0.005. Shoot dry weight. RWC: Relative water content. EL: Electrolytes leakage. MDA: Malondialdehyde. TSS: Total soluble sugars. CAT: Catalase activity. APX: Ascorbate peroxidase activity.

### 3.4 Principal component analysis (PCA)

PCA was used as a multivariate statistical tool to represent the variability of physiological parameters of different cultivars studied under drought stress. This analysis showed three top components (Eigen value ≥ 1), accounting for 86.45% of the total variation of the physiological parameters under drought stress. PC1, PC2, and PC3 explained, respectively, 39.12%, 33.37%, and 13.96% of the total variation.

**Fig. 5:**
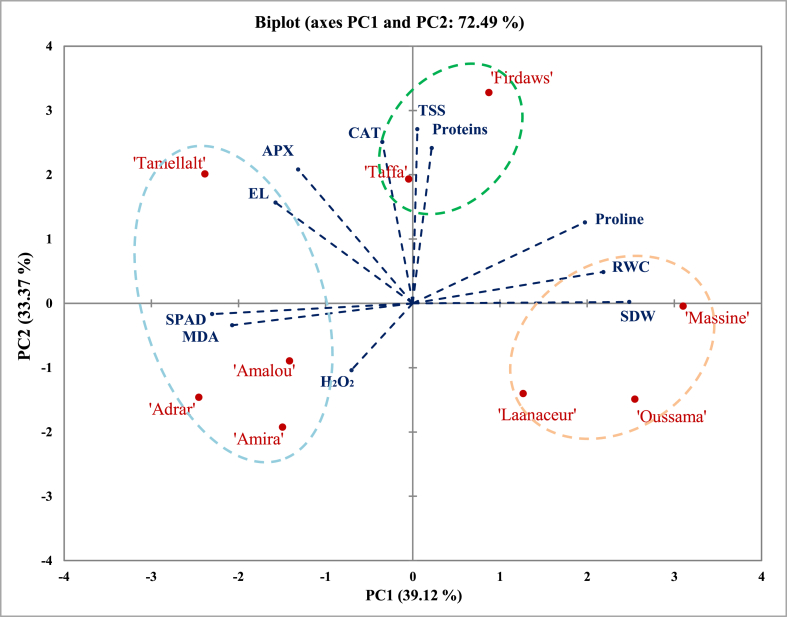
Biplot of principal component analysis for physiological responses in nine barley Moroccan cultivars. Shoot dry weight. RWC: Relative water content. EL: Electrolytes leakage. MDA: Malondialdehyde. TSS: Total soluble sugars. CAT: Catalase activity. APX: Ascorbate peroxidase activity.

To select tolerant cultivars, a biplot was created using the two first principal components (Fig. 5). The negative side of PC1 showed ‘Adrar’, ‘Amalou’, and ‘Amira’ with high scores of SPAD value and MDA content. The positive side showed ‘Massine’ and ‘Oussama’ with high values of SDW, RWC, and proline content. The positive side of PC2 discriminated ‘Tamellalt’ with a high value of EL% and APX activity, while ‘Firdaws’ and ‘Taffa’ were discriminated with high levels of TSS and proteins contents, and CAT activity.

### 3.5 Agglomerative hierarchical cluster analysis

**Fig. 6:**
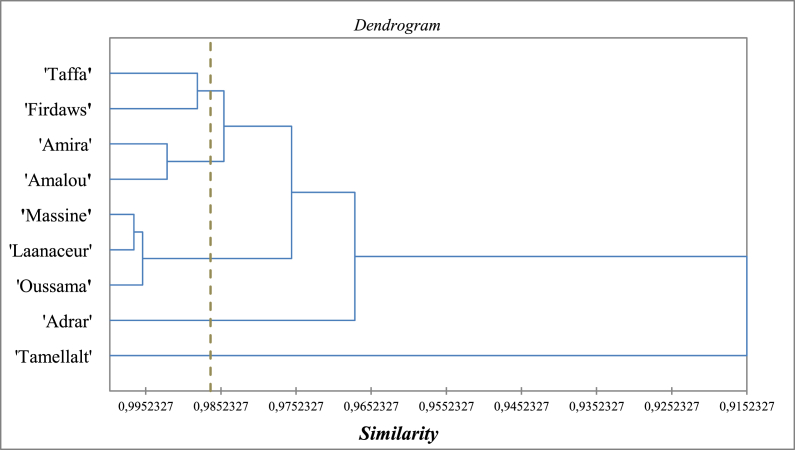
Dendrogram for agglomerative hierarchical cluster analysis based on physiological characteristics under drought stress of leaves of nine barley Moroccan cultivars.

Values of leaves' biochemical and physiological parameters under drought stress can be used to classify barley cultivars by their tolerance degree to drought stress. This analysis is based on regrouping the cultivars with similar biochemical and physiological characteristics under drought stress into the same cluster. Cluster analysis for the nine Moroccan barley cultivars divided them into 5 classes, as shown in the dendrogram (Fig. 6). ‘Taffa’ and ‘Firdaws’ were grouped in the first class with 98.6% similarity. ‘Amira’ and ‘Amalou’ were classified in the second class with 99% similarity. While ‘Massine’, ‘Laanaceur’, and ‘Oussama’ were classified in the third class with 99.5% of similarity, ‘Adrar’ and ‘Tamellalt’ were classified alone in the fourth and fifth classes.

## 4 Discussion

Abiotic stresses affect plant growth and crops production [42] by various ways. Especially by generating ROS and osmotic stress [32], which leads to damage plant cells steadiness [43,44]. However, To combat stress, plants have developed enzymatic antioxidants like SOD, CAT, and GPX … [33] as well as non-enzymatic antioxidants with high radical quenching capacities like betalain, betacyanin, betaxanthin, chlorophyll pigments, phenolic, and flavonoids … [[45], [46], [47], [48], [49], [50], [51], [52]], Plants subjected to abiotic stress developed defense mechanisms to increase the content of these antioxidants [53] and detoxify ROS.

Drought stress is the most prevalent and prominent abiotic stress influencing plants’ growth and development [2]. In cereals, water deficits affect many morphological, biochemical, and physiological parameters. Leaf and root growth are inhibited under moderate and severe stress [54]. Drought stress affects photosynthesis, water relations, nutrient uptake, oxidative status, osmotic balance, and hormonal balance, which impact the yield [3,19]. Therefore, to ensure food security, the selection of tolerant cultivars has become indispensable. In this study, Moroccan cultivars of barley (*Hordeum vulgare* L.) were screened for physiological and biochemical characteristics to select cultivars that are able to cope with drought stress.

The results showed significant intraspecific variability regarding the adaptation to drought conditions, as reported in many papers studying barley growth under drought stress [9,21,55]. The preservation of a high SDW value under stressful conditions is primarily related to high stress tolerance. The reduction of SDW under osmotic stresses is mainly explained by economization of plants resources by arresting cell proliferation and subsequent cell expansion [3,56]. In our case, ‘Firdaws’, ‘Laanaceur’, ‘Massine’ and ‘Oussama’ were less affected compared to the other cultivars, which makes them more resistant against deficit water conditions based on this trait. RWC is a critical physiological criterion for determining the degree of tissue and cell hydration required for optimal physiological growth and functioning in plants [57,58]. Preservation of a high value of RWC under drought stress indicates an important drought tolerance [27,59]. In our results, a significant reduction in RWC was noticed under drought stress in the leaves of barley cultivars. Similar results were reported in other studies when barley plants were exposed to water deficits [[11], [12], [13], [14]]. As shown in Fig. 1b, RWC of ‘Adrar’, ‘Amalou’, ‘Amira’, ‘Firdaws’, and ‘Tamellalt’ are the most influenced by drought stress. This means that these cultivars are less able to keep cell turgor in their leaves under drought stress. However, ‘Laanaceur’, ‘Massine’, ‘Oussama’, and ‘Taffa’ are less influenced, meaning high capability of these cultivars to keep cells turgor in their leaves under drought conditions.

Drought stress induced a significant decrease in chlorophyll content (SPAD value), as shown in Fig. 1d. Such results were also reported by other studies dealing with cereals [58,60,61]. Chlorophyll content reduction may be due to electrolyte leakage from thylakoïds membrane and lipids peroxidation [58], protoplasm dehydration, and less photo-assimilation level [10]. The increase of electrolyte leakage in leaf tissues is considered as an index of membrane damage and deterioration [62] and as the parameter indicating membrane steadiness [63]. The increase of EL percentage under stress conditions is always associated with the sensitivity of the plant to oxidative stress [28]. In our findings, different levels of increase in EL% were noted, which is in agreement with the literature [10,15]. As presented in Fig. 1c, ‘Tamellalt’ cultivar was the most influenced by water deficit regarding electrolyte leakage, followed by ‘Fidraws’, ‘Taffa’, ‘Amira’, and ‘Adrar’. This indicates membrane cell damage in the tissues of those cultivars under drought stress. However, for the other cultivars, there was no significant impact of drought stress on their EL%, indicating an important cell membrane steadiness under drought stress and high tolerance.

Under drought conditions and other abiotic stresses, the accumulation of proline is common in most cereals [10,19,61,64]. This molecule is an important osmoregulator for membrane stability, buffering cellular redox potential and scavenging free radicals [19]. Proline can also play an important role in the activation of the detoxification pathway [65], making cultivars with the ability to produce more proline under stress more tolerant. The cultivars ‘Firdaws’, ‘Laanaceur’, ‘Massine’, and ‘Oussama’ accumulated the highest levels of proline under drought stress (Fig. 2a), elucidating that these cultivars are more resistant to drought stress. The accumulation of proline is widely reported as a known response in stress tolerant plants. Many roles have been attributed to this molecule regarding its involvement in plant tolerance to abiotic stress, but the exact mode of its action is still unclear. It is also important to keep in mind that high levels of proline could have a negative impact on plant cells. The ability to maintain the intracellular proline content in balance seems to be essential for plant tolerance and survival [66]. Actually, proline levels in the cell are determined by the balance between biosynthesis and catabolism. Proline is synthesized from ornithine and glutamate, the glutamate pathway being more predominant in the plant cell [66]. On the other hand, the level of proline content in the cell is regulated by the actions of proline dehydrogenase (proDH) and pyrroline 5-carboxylate dehydrogenase (P5CDH). These enzymes are transcriptionally regulated by developmental and environmental signals, and proline catabolism is enhanced during the recovery period from stress [67]. The level of accumulation of proline is reported to vary markedly between species and between genotypes within species [68,69]. This could be linked to the complexity of the signaling networks and the multigenic processes involved in the regulation of the proline biosnynthesis pathway and its accumulation in plant cells under environmental constraints [70].

As shown in Fig. 2b, except for ‘Amalou’, ‘Amira’, and ‘Massine’, the other cultivars recorded an increase in TSS content. For protein content, a great increase was marked under drought stress in all cultivars (Fig. 2c). This is in agreement with literature results where tolerant barley cultivars accumulate sugars and proteins in their leaves under drought stress [28]. The accumulation of soluble sugars and proteins is associated with the osmotic regulation of cells. TSS and protein accumulation decrease osmotic potential inside the cell, making cells able to keep a high turgor potential [28,71], and stabilize the cell membrane by reacting with the lipid bilayer [21].

Based on biochemical, molecular, and genetic findings, it has been determined that soluble sugars play a critical role in the web regulation of plants’ adaptation against biotic and abiotic stresses [72]. In recent years, sugars have been more studied for their hormone-like functions as a primary messenger in signal transduction [73]. Glucose is widely known for its role as a modulator in the repression of genes implicated in ABA catabolism and the activation of genes implicated in ABA biosynthesis [74]. It is also proved that high levels of sugar lead to the repression of the genes implicated in photosynthesis [75]. Indeed, it is well documented that the repression of the Rubisco small subunit (RBCS) gene is associated with high sugar concentration in potatoes and maize [72,76]. Furthermore, sugars accumulation is linked with down regulation of many genes implicated in photosynthesis such as atp-δ thylakoid ATPase (ATP- δ) gene, chlorophyll *a*/b binding protein (CAB) gene, pyruvate phospho dikinase (PPDK) gene, C4 malic enzyme gene (ME1) and C4 PEP carboxylase (PEPC1) gene [72]. Hu et al. [77] showed that proline accumulation varies increasingly with glucose concentration applied to wheat plants under salt stress. This can help plants to be more tolerant to abiotic stress by increasing the content of proline having the functions cited above.

Under drought conditions, a significant increase in MDA and hydrogen peroxide content in leaves was observed (Fig. 4). This was also the case in other studies [20,21]. Various environmental stresses including drought constraints, induce the generation of reactive oxygen species (ROS), which can lead to the oxidation of DNA and proteins, the peroxidation of membrane lipids, hydrogen peroxide accumulation, and oxidative burst cell [29,78]. On the other hand, lipid peroxidation could be triggered by increased lipoxygenase activity [19,79]. Both enzymatic and non-enzymatic processes are reported to be involved in the formation of lipid peroxidation products in plants such as MDA and jasmonates under oxidative stress conditions [80]. In cereals, low H_2_O_2_ and MDA contents were associated with high stress tolerance ability [19]. Actually, the role of aldehyde compounds (MDA) produced under stress, environmental or developmental signals depends upon their accumulation levels which are controlled by the balance between the lipid peroxidation intensity and the activity of aldehyde dehydrogenases (ALDHs). It is well established that the expression and the activity of ALDHs are induced by H_2_O_2_, abscissic acid, and MDA [81]. These molecules could serve as signals of protection processes where ALDHs contribute to maintaining the cellular redox homeostasis and reducing potential NADPH required for antioxidant activity of the ascorbate-glutathione cycle and photosynthesis process [82,83]. In this study, ‘Massine’, ‘Oussama’, and ‘Tamellalt’ cultivars were characterized both by lower values of H_2_O_2_ and MDA content compared with other cultivars. This makes them less affected by ROS and/or lipoxygenase under drought conditions. The lower content of MDA in plant cells could be interpreted as a defense mechanism for signaling rather than an indicator of membrane damage and protein carbonylation [80]. When MDA is accumulated in the cells at a high level, proteins are carbonylated, such as PSII core proteins and Rubisco, leading to disturbances in all plant cell metabolism which may trigger cell death. All previously published results converge towards the synthesis and involvement of MDA in plant metabolism under environmental stress. However, the role of this molecule remains unclear and needs more investigation.

Owing to their ability to scavenge free radicals and ROS, the expression levels of CAT and APX were investigated in irrigated and stressed barley plants. Our results (Fig. 3) showed a significant increase in the activities these two enzymes in most cultivars studied under drought stress. These findings are supported by various other studies [2,30]. Among these antioxidant enzymes, CAT correlates significantly and negatively with H_2_O_2_ content, which is in the same way with many other papers [84]. This negative correlation might be explained by an increased role of CAT in H_2_O_2_ detoxification under stressful conditions [84]. On the other hand, there was no correlation between APX and hydrogen peroxide content, which might indicate that the intervention of CAT in the ROS detoxification pathway is more important than APX [84]. Taken together, it may be suggested that plants' tolerance against osmotic stresses varies increasingly according to up-regulation of these enzymes' expression, especially CAT activity. Based on these results, it seems that ‘Adrar’, ‘Amalou’, ‘Firdaws’, and ‘Massine’ cultivars significantly increase CAT and APX activities under drought stress, which makes the enzyme machinery of these cultivars more able to react to drought stress compared to other cultivars studied.

It is widespread in the literature that drought tolerance is associated physiologically with high values of SDW, RWC, antioxidants enzyme activities, and proline content [19,57,58,85]. In our results, the biplot (Fig. 5) discriminated ‘Massine’ and ‘Oussama’ as the two cultivars with the highest levels of SDW and RWC. Indeed, the agglomerative hierarchical cluster analysis (Fig. 6) classified these cultivars with ‘Laanaceur’ in the third class considered, including cultivars showing high physiological tolerance against drought stress. Furthermore, high scores of H_2_O_2_, MDA contents [[19], [20], [21]], and EL% [10,62,63] were always considered as signs of plants' sensitivity against abiotic stresses. The biplot of our results (Fig. 5) discriminated ‘Tamellalt’ with a high percentage of electrolyte leakage, ‘Adrar’, ‘Amalou’ and ‘Amira’ with high values of MDA and H_2_O_2_ content. Except for ‘Tamellalt’, using agglomerative hierarchical cluster analysis, these cultivars were classified into the first and second classes of considered describing sensitive cultivars against drought stress. ‘Tamellalt’ was classified alone in the last class as the most sensitive cultivar (Fig. 6). Khattabi et al. (2022) studied the sensitivity of six of these cultivars to salt stress. The results showed a relative resistance in ‘Laanaceur’, ‘Taffa’, and ‘Tamellalet’, while ‘Oussama’ and ‘Amira’ were described as salt sensitive cultivars.

## 5 Conclusions

In this study, we screened physiological and biochemical traits in barley leave of nine Moroccan cultivars grown under drought stress. A remarkable variability was recorded between cultivars. A significant impact (P < 0.05) of drought stress with different levels is described in our results. Drought treatment induced a significant reduction of SDW, RWC, and chlorophyll content (SPAD index), accumulation of proline, an increase of hydrogen peroxide, MDA, soluble sugars, and proteins contents in leaves of barley cultivars, and also a significant increase of antioxidant enzymes activities (CAT and APX) ‘Firdaws’, ‘Laanaceur’, ‘Massine’, ‘Oussama’ and ‘Taffa’ maintained high values of SDW, RWC, and proline content, and also increased CAT and APX activities under drought conditions, which could be linked to their drought tolerance. ‘Adrar’, ‘Amalou’, ‘Amira’, and ‘Tamellalt’ showed high values of MDA and H_2_O_2_ content which indicates drought sensitivity of these barley cultivars. Further assessments is needed to evaluate the drought effect on field grain stage, phenology, grain quantity, and quality in these Moroccan cultivars.

## Author contribution statement

Mohamed Ferioun, PhD student: Conceived and designed the experiments; Performed the experiments; Analyzed and interpreted the data; Contributed reagents, materials, analysis tools or data; Wrote the paper.

Nassira Srhiouar; Said Bouhraoua: Performed the experiments.

Naima Elghachtouli; Said Louahlia: Conceived and designed the experiments; Analyzed and interpreted the data; Contributed reagents, materials, analysis tools or data.

## Funding statement

This research did not receive any specific grant from funding agencies in the public, commercial, or not-for-profit sectors.

## Data availability statement

No data was used for the research described in the article.

## Declaration of interest's statement

The authors declare that they have no known competing financial interests or personal relationships that could have appeared to influence the work reported in this paper.
