# Expression of concern: “Physiological and biochemical changes in Moroccan barley (*Hordeum vulgare* L.) cultivars submitted to drought stress” [Heliyon 9 (2023) e13643]

**DOI:** 10.1016/j.heliyon.2025.e43816

**Published:** 2025-09-06

An investigation conducted on behalf of the journal by Elsevier's Research Integrity & Publishing Ethics team has determined that the authors were requested by 2 of the reviewers to insert redundant references to their paper during the peer-review process. As these citations were requested by individuals who could influence the decision to accept the article, the authors are not considered at fault for the addition of these citations. The Editors have determined that the citations added to the paper at the request of the reviewers does not offer a balanced reflection of the literature. Readers should take this into consideration when evaluating this paper.

